# Improving the Efficiency of Photodynamic Chemotherapy in Root Canals against *Enterococcus faecalis* In Vitro

**DOI:** 10.3390/antibiotics9090543

**Published:** 2020-08-26

**Authors:** Christian Tennert, Yoana Zinovieva, Kalin Shishkov, Lamprini Karygianni, Makus Jörg Altenburger, Richard J Wierichs, Ali Al-Ahmad

**Affiliations:** 1 Department of Restorative, Preventive and Pediatric Dentistry, University of Bern Bern, Freiburgstrasse 7, 3007 Bern, Switzerland; Richard.wierichs@zmk.unibe.ch; 2 Faculty of Medicine, Department of Operative Dentistry and Periodontology, University of Freiburg—Medical Center, Hugstetter Str. 55, 79106 Freiburg, Germany; yoana.zinovieva@gmail.com (Y.Z.); kalin.shishkov@gmail.com (K.S.); Markus.altenburger@uniklinik-freiburg.de (M.J.A.); Ali.al-ahmad@uniklinik-freiburg.de (A.A.-A.); 3 Clinic of Preventive Dentistry, Periodontology and Cariology, Center of Dental medicine, University of Zürich, 8006 Zürich, Switzerland; lamprini.karygianni@zzm.uzh.ch

**Keywords:** endodontics, antimicrobial treatment, photodynamic therapy, isopropanol, *Enterococcus faecalis*

## Abstract

The aim of this study was to evaluate the effect of photoactivated chemotherapy (PACT) on *Enterococcus faecalis* (*E. faecalis*) biofilms in root canals using an 90% isopropanol (IPA)-based photosensitizer and removing excess photosensitizer before light incubation. Three hundred and seven extracted human teeth with one root canal were infected with *E. faecalis* for 72 h and treated in groups: IPA irrigation; PACT; PACT and final rinse with IPA; PACT with photosensitizer removal using either 0.9% NaCl solution or sterile paper points or both; PACT using IPA-based photosensitizer with and without a final rinse of IPA. Root canals were sampled using sterile paper points and dentin chips collected from the root canal walls. Additionally, SEM (Scanning Electron Microscopy) images of the specimens were taken to evaluate the root canal walls for residue bacterial contamination. In all antimicrobial treatment groups treatments *E. faecalis* counts were significantly reduced in the root canals. Using IPA-based photosensitizer the antimicrobial effect of PACT was significantly enhanced. Irrigation with IPA alone or after PACT significantly increased the antimicrobial effect compared to PACT alone. The collected dentin chips revealed the highest amount of culture negative root canals (10%) after PACT using IPA-based photosensitizer. In the other groups, the culture negative samples ranged from only 0–2 specimens of 30 specimens. REM images show remaining *E. faecalis* cells on the root canal wall and inside dentin tubules. Using IPA-based photosensitizer significantly enhanced the antimicrobial effect of PACT against *E. faecalis* in the root canals.

## 1. Introduction

The aim of endodontic therapy is to eliminate as many microbiota as possible from the root canal system by chemomechanical preparation of the root canal system [1]. Apical periodontitis after root canal treatment is mostly associated with remaining microorganisms inside the root canal system causing an infection of the root surrounding tissues [1,2]. *Enterococcus faecalis* (*E. faecalis*) is frequently found in teeth with persistent periradicular infections [3,4,5]. Previous studies found *E. faecalis* to be associated with endodontic post-treatment disease [3,4,5]. In cases of failed root canal treatments, oral *E. faecalis* strains have been isolated in up to 77% [6]. Chemomechanical root canal preparation is performed using endodontic files and irrigants. There are various endodontic irrigants on the marked providing antimicrobial and biofilm-dissolving effects (Zehnder 2006). Besides sodium hypochlorite and chelating agents, antimicrobial effects have been demonstrated for isopropanol (IPA) in earlier studies [7,8,9,10]. Although chemomechanical preparation of root canals, nearly 40–60% of root canals contain culturable microbial species [1,11,12]. Even if passive sonic and ultrasonic irrigation have been demonstrated to be effective in reducing *E. faecalis*, it is a very resistant microbial species in endodontic infections [13]. In particular, *E. faecalis* has been found to be very resistant against antimicrobial procedures in endodontics, e.g., intracanal dressings, like calcium hydroxide and antimicrobial irrigants, such as NaOCl and chlorhexidine and even tetracycline [14,15,16].

Photoactivated chemotherapy (PACT) is a treatment method that utilizes the activation of a dye (photosensitizing agent, photosensitizer) that is exposed to light of a specific wavelength in the presence of oxygen [17,18,19]. Previous studies showed that PACT alone is able to significantly reduce microorganisms in root canals in vitro [20,21,22]. However, in several in vitro reports the antimicrobial efficacy against *E. faecalis* in root canals of PACT was lower compared to NaOCl irrigation [23,24,25,26,27,28]. In an animal study, treating teeth with experimentally induced apical periodontitis in one session endodontic treatments, PACT was found to be a promising adjunctive antimicrobial therapy [29]. In cases specifically with persistent *E. faecalis* infection or other drug-resistant species, PACT seems to be a promising adjunct for the elimination of these microbiota [25,28,30]. In previous in vitro studies using root canals artificially infected with *E. faecalis*, the influence of ultrasonic activation of the photosensitizers and modifications of the photosensitizers were investigated [31,32]. The authors found that PACT using ultrasonic activation of the photosensitizer or modified photosensitizers reduced *E. faecalis* to a significantly greater extend compared to conventional PACT. However, PACT using modified photosensitizers could not completely eradicate *E. faecalis* and was less effective than disinfecting agents, e.g., NaOCl. Modifications of the photosensitizers were able to enhance the antimicrobial effect of PACT. Therefore, the present study investigated, whether PACT using modified photosensitizer with 90% isopropanol (IPA) as a solvent for the dye or modifications of the protocol in PACT application by removing access photosensitizer using 0.9% NaCl and/or sterile paper points may have a greater antimicrobial effect on *E. faecalis* biofilm-like structures in the root canals. The hypothesis of this study was that the modified photosensitizer or the modifications of PACT applications by removing access photosensitizer before light activation are able to enhance the antimicrobial effect of PACT.

## 2. Materials & Methods

### 2.1. Tooth Specimens

Intact permanent human teeth with one root and one root canal (mandibular and maxillary front teeth and premolars) were used. Patients gave their written informed consent for using the extracted teeth for research. The study protocol was reviewed and approved by the ethics committee of the University of Freiburg (604/14). The characteristics and visual and radiographic examination of the collected teeth used for this investigation is described in a previous study from our research group [32].

A total of three hundred and seven (9 groups of 33 specimens + 10 untreated control) were prepared as described previously [28,32].

Standard access cavities were prepared to completely access the pulp chamber. The root canal was instrumented using a hand file (size ISO 10, K-type, VDW, Munich, Germany) and apical patency through the apical foramen was confirmed. The tooth length was determined with an ISO size 10 file becoming visible at the apical foramen using 5× magnification (magnifying glasses, Carl Zeiss Jena GmbH, Jena, Germany). The working length (WL) was set 1 mm shorter than tooth length. Each root canal was mechanically prepared using ProTaper Universal instruments (Dentsply Sirona, Bensheim, Germany) with an Endo IT Professional motor (VDW, Munich, Germany) at 250 rpm. The preparation of the root canals was performed using ProTaper S1 and S2 shaping files and F1 and F2 finishing files according to the manufacturer’s instructions. For irrigation, a 3% NaOCl solution was used during chemomechanical preparation. Patency was maintained by repeatedly inserting an ISO size 10 nickel–titanium K-type file. After chemomechanical preparation, root canals were dried with paper points. After chemomechanical preparation, the teeth were embedded in methacrylate (Techovit 4070™, Haereus Kulzer^®^, Wernheim, Germany) and decoronated at the cementum–enamel junction as described previously [32]. After a final rinse of the root canals using 5 mL of 3% NaOCl, 2.5 mL of 20% EDTA (pharmacy University Medical Center Freiburg) and 5% sodium thiosulfate (Na_2_S_2_O_3_) solution, the specimens were autoclaved at 134 °C for 18 min. To verify that root canals were free of any culturable microorganisms after autoclaving, a sample was taken from each root canal with three sterile ProTaper paper points (size F2, Dentsply Sirona, Bensheim, Germany). The paper points were transferred into 750 µL of sterile 0.9% NaCl solution, vortexed for 30 s and 100 µL were cultured on a blood agar plate. Teeth with a positive bacterial culture were excluded. This procedure was performed as described in a previous study [32].

### 2.2. Infection of Specimens and Antibacterial Treatment

The root canals of the specimens were infected with a clinical isolate of *Enterococcus faecalis* (*E. faecalis*) T9 (ATCC 1943) as described previously [28,32]. *E. faecalis* was cultivated for 72 h in the root canals of the specimens to achieve biofilm formation on the root canal walls. Root canals were then rinsed with 2.5 mL of sterile 0.9% NaCl solution. To determine the contamination of the root canals, 3 sterile paper points (ProTaper, size F2, Dentsply Sirona, Bensheim, Germany) were used to take a baseline sample. The paper points were transferred into 750 µL of sterile 0.9% NaCl solution. After vortexing for 30 s, 100 µL were cultured on Columbia blood agar (CBA) plates and colony-forming units (CFU) of *E. faecalis* were counted.

In an untreated control group (10 specimens), only sterile TSB was syringed into the root canals of the specimens. After 72 h of incubation, samples were taken using 3 sterile paper points (ProTaper, size F2, Dentsply Sirona, Bensheim, Germany) and cultured on CBA to verify that the root canals of these specimens were free from any microorganisms.

The specimens were randomly divided into nine different treatment groups. The first three groups served as controls.

In the first group (*n* = 30), root canals were rinsed with 5 mL of 0.9% NaCl solution.

In the second group (*n* = 30), the root canals were treated by irrigation using 5 mL of 90% IPA solution.

In the third group (*n* = 30), root canals were treated using conventional PACT with an NaCl-based photosensitizer. Toluidine blue (TBO) (16 µg/mL, diluted in sterile 0.9% NaCl) solution was used as photosensitizer. A syringe and a 30 gauge needle were used to insert the photosensitizer solution into the root canal. After 60 s of incubation, the photosensitizer solution was activated for 2 ms using the PACT Endo tip (PACT, Cumdente, Tübingen, Germany, 100-mW light-emitting diode (LED), wavelength of 635 nm) according to the manufacturer’s instructions.

In the fourth group (*n* = 30), root canals were treated as described in the previous group. Additionally, the root canals were irrigated using 2.5 mL of 90% isopropanol (IPA) after PACT treatment.

In the fifth group (*n* = 30), a modified TBO solution (16 µg/mL, diluted in 90% IPA) was used as photosensitizer. The photosensitizer solution was syringed into the root canal, incubated and light activated as described above according to the manufacturer’s instructions.

In the sixth group (*n* = 30), a modified TBO solution (16 µg/mL, diluted in 90% IPA) was used as photosensitizer. The photosensitizer solution was syringed into the root canal, incubated and light activated as described above. Thereafter, the root canals were irrigated using 2.5 mL of 90% IPA.

In the seventh group (*n* = 30), TBO (16 µg/mL, diluted in sterile 0.9% NaCl) was used as photosensitizer and syringed into the root canal as described above. After 60 s of incubation, the root canals were then irrigated using 2.5 mL of 0.9% NaCl solution to remove the photosensitizer solution. Light activation was performed using the PACT light source as described above.

In the eighth group (*n* = 30), TBO (16 µg/mL, diluted in sterile 0.9% NaCl) was used as photosensitizer and syringed into the root canal as described above. After 60 s of incubation, the photosensitizer solution was removed using sterile paper points (ISO 25, Dentsply Sirona, Bensheim, Germany). Light activation was performed as described above according to the manufacturer’s instructions.

In the ninth group (*n* = 30), TBO (16 µg/mL) diluted in sterile 0.9% NaCl solution was used as photosensitizer and syringed into the root canal as described above. The photosensitizer solution was syringed into the root canals and incubated as described above. The photosensitizer solution was removed by irrigation using 2.5 mL of 0.9% NaCl solution and drying the root canals with sterile paper points (ISO 25, Dentsply Sirona, Bensheim, Germany). Light activation was performed using the PACT light source as described above.

After antimicrobial treatments, all specimens of the different treatment groups were irrigated using 2.5 mL of sterile 0.9% NaCl solution and sampled using three sterile paper points (size F2, Dentsply Sirona, Bensheim, Germany). The paper points were transferred into 750 µL of sterile 0.9% NaCl solution and vortexed for 30 s. Therof, 100 µL were brought on blood agar plates, cultured and CFUs were counted.

After the specimens were sampled, another sample of each root canal was taken using a sterile ProTaper F3 hand file. The root canals were instrumented to remove dentin chips from the root canal wall. Thereafter, 40 µL of sterile 0.9% NaCl solution were brought into each root canal using a 30 gauge needle. Each root canal was then sampled again using three sterile paper points (size F3, Dentsply Sirona, Bensheim, Germany). The cutting part of the hand file was cut and also collected with each sample in a vial containing 750 µL of sterile 0.9% NaCl solution. After vortexing each sample for 30 s, 100 µL thereof were cultured on CBA plates. Culturing of the samples was performed as described in a previous study [33]. A dilution series was performed up to 10^−5^ for each sample. The dilutions were cultured on CBA Plates at 37 °C and 5–10% CO_2_ atmosphere for 24 h an CFUs per mL were counted.

### 2.3. Scanning Electron Microscopy (SEM) Imaging of the Specimens

For SEM imaging the root canals of the specimens were prepared as described above. Using a rotary cutting diamond saw, the specimens were cut vertically in two pieces. The pieces of the tooth specimens were autoclaved at 134 °C for 18 min. *E. faecalis* infection of the specimen sections was performed in TSB in chambered coverslips (µ-Slide 8 well, ibidi GmbH, Martinsried, Germany) for 72 h. TSB was replaced with fresh TSB every day. Thereafter, antimicrobial treatment of the root canals was performed in nine different groups (*n* = 3 in each group) as described above. The specimens were randomly divided into the different treatment groups and treated as described above. Then, root canals were irrigated using 1 mL of sterile 0.9% NaCl solution and the cover slip was removed. The specimens were fixed in 8% formaldehyde overnight at 4 °C and dehydrated in increasing alcohol concentrations (30%, 50%, 70%, 80%, 90% once each and twice in 99.8% for one hour). Afterwards, the specimens were critical point dried (critical point dryer CPD 030; Bal-Tec, Wallruf, Germany) using liquid carbon dioxide. The specimens were subsequently sputtered with gold in an SCD 050 coater (Bal-Tec) and the root canal walls were examined using a Zeiss Leo 435 VP scanning electron microscope (Leo Electron Microscopy, Ltd. Cooperation Zeiss Leica, Cambridge, England) at 10–12 kV. This procedure was performed as described in a previous study [32].

### 2.4. Statistical Analysis

For descriptive analysis mean and standard deviation were computed. For graphic presentation boxplots were used.

Linear regression models were applied to compare the differences between the treatment groups, the baseline value was used for adjustment. Student–Newman–Keuls methods were used to correct for the problem of multiple testing (adjustment of *p*-values). To prove a group difference of the percentage change in 2 groups of 4% (SD 4) with a power of 90%, 23 subjects per group are needed. Since several comparisons and thus a correction for multiple testing were planned, a case number of 30 per group was chosen.

The statistical software STATA 14.2 (StataCorp, College Station, TX, USA) was used to perform the calculations.

## 3. Results

### 3.1. Antimicrobial Effect of the Treatments on Enterococcus Faecalis

**Figure 1:**
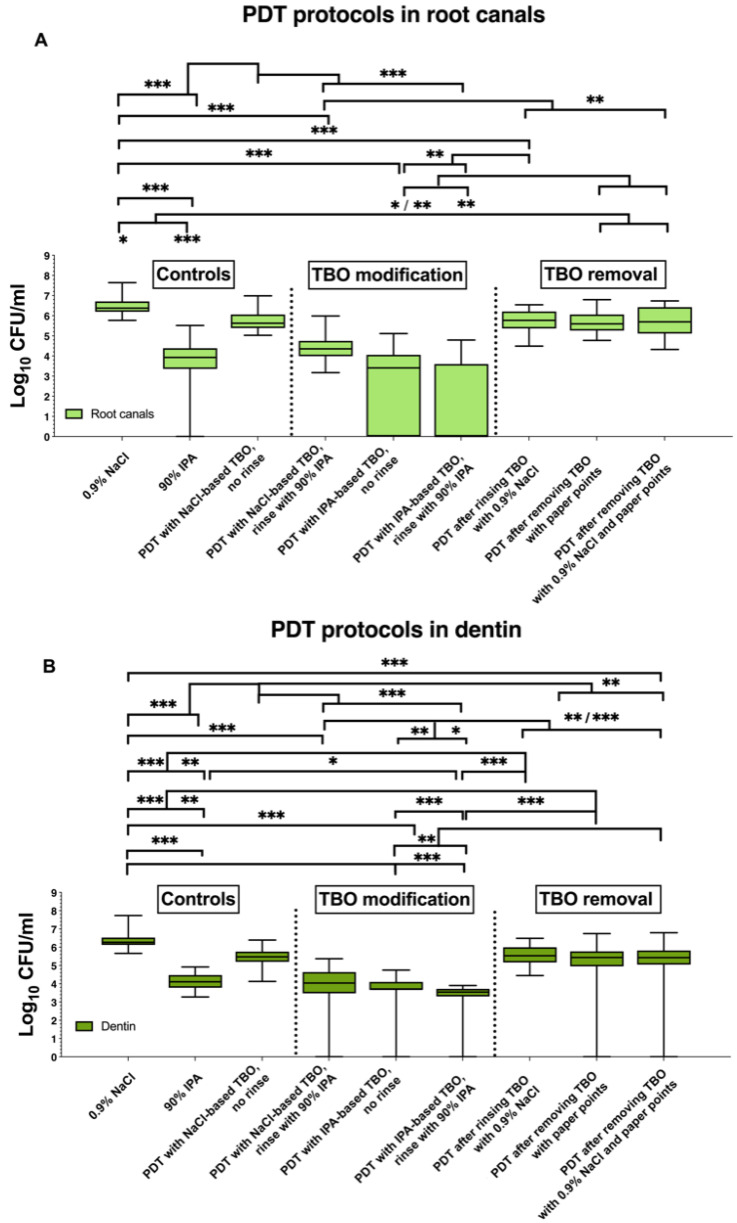
Boxplots of colony-forming units (CFU) counts, demonstrating the antimicrobial effect of PACT on *E. faecalis* after 72 h-incubation in root canals of extracted teeth (*n* = 270). After antimicrobial treatment, root canals were sampled using (**A**) sterile paper points and (**B**) collecting dentin chips. Toluidine blue (TBO, 16 µg/mL) served as the photosensitizer, activated by a 100-mW LED light source. The following treatment groups were tested: irrigation using 0.9% NaCl (negative control) or 90% isopropanol (IPA; positive control), conventional photoactivated chemotherapy (PACT) with NaCl-based TBO and no final rinse (standard control), PACT with NaCl-based TBO and final rinse with 90% IPA, PACT with IPA-based TBO and no final rinse, PACT with IPA-based TBO and final rinse with 90% IPA, PACT with removal of access photosensitizer using either 0.9% NaCl solution or sterile paper points or both. Box plots represent the CFUs determined by selective agar plating, while horizontal lines indicate their median values. Undetectable values were ascribed the lowest detection limit value of the assay to allow for log transformation. The CFUs are presented on a log_10_ scale per mL (log_10_/mL). The *p*-values of the significantly different data are marked on the graphs (*, *p* ≤ 0.05; **, *p* ≤ 0.01; ***, *p* ≤ 0.001).

**Figure 2:**
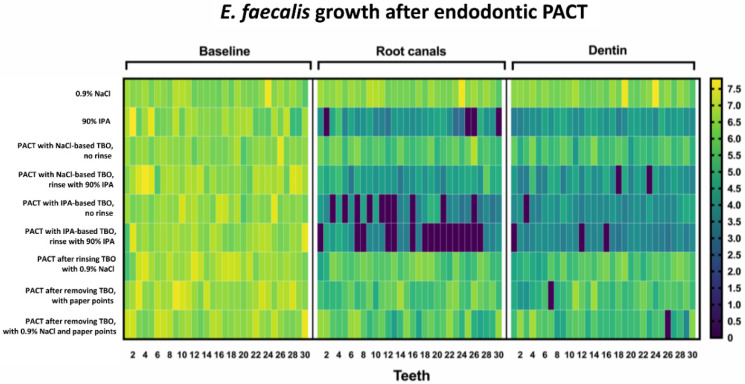
Heatmap showing the absolute distribution (in log_10_/mL) of *E. faecalis* after 72 h-incubation and subsequent PACT in root canals of extracted teeth (*n* = 270). Samples taken prior to treatment served as baseline, while samples from root canals and dentin chips were collected after PACT treatment. A 16-µg/mL toluidine blue solution (TBO) served as the photosensitizer, activated by a 100-mW LED light source. The following treatment groups were tested: irrigation using 0.9% NaCl (negative control) or 90% isopropanol (IPA; positive control), conventional PACT with NaCl-based TBO and no final rinse (standard control), PACT with NaCl-based TBO and final rinse with 90% IPA, PACT with IPA-based TBO and no final rinse, PACT with IPA-based TBO and final rinse with 90% IPA, PACT with removal of access photosensitizer using either 0.9% NaCl solution or sterile paper points or both. Tooth numbers for each treatment group are shown in columns and treatment groups in rows. The colors as depicted on the color scale bars on the right vary to indicate data values change from very low to extremely high.

Overall, the different antimicrobial treatments significantly reduced the contamination of the root canals with *Enterococcus faecalis* (*E. faecalis*) (Figure S1). Comparing the contamination of specimens of the different groups before antimicrobial treatment, there were no statistically significant differences between the baseline samples. Mean CFU levels after treatment were the highest for irrigation using the 0.9% NaCl solution. The lowest antimicrobial effect was found for conventional PACT treatment using 0.9% NaCl-based TBO. Rinsing root canals of the specimens with 90% IPA reduced *E. faecalis* counts by 98.8%. Conventional PACT treatment using an NaCl-based photosensitizer followed by irrigation with 90% IPA reduced *E. faecalis* counts by 99.2%. Treatment of root canals with PACT using IPA as a solvent for the photosensitizer resulted in a reduction in bacterial load by 99.3% and the additional irrigation with 90% IPA after PACT with toluidine blue dissolved in 90% IPA resulted in a 99.9% elimination of *E. faecalis*. According to intragroup analysis, the lowest survival rates of *E. faecalis* was found in the groups using 90% IPA either as irrigants after conventional PACT with the NaCl-based photosensitizer or in the groups using IPA as solvent for the photosensitizer for PACT treatment, as well as after irrigation with 90% IPA alone (Figure 1 and Supplementary Figure S2). In these groups, a reduction of *E. faecalis* counts of more than 98% was achieved and was found to be statistically significant (*p* < 0.001) compared to the other treatment groups. Analyzing the samples of the collected dentin chips using the ProTaper F3 hand file, PACT using toluidine blue dissolved in 90% IPA and the additional irrigation with 90% IPA achieved culture negative canals in 3 of 30 cases (10%). In the other groups culture negative samples ranged from only 0–2 specimens of 30 specimens (Figure 1). The heat map (Figure 2) displays the contamination of each specimen separately. In treatment groups using 90% IPA as an irrigant or PACT with the IPA based photosensitizer the remaining contamination of the root canal and particular the root canal dentin was lower compared to the other treatment groups.

### 3.2. Scanning Electron Microscopic Evaluation of Root Canals after Antimicrobial Treatment

**Figure 3:**
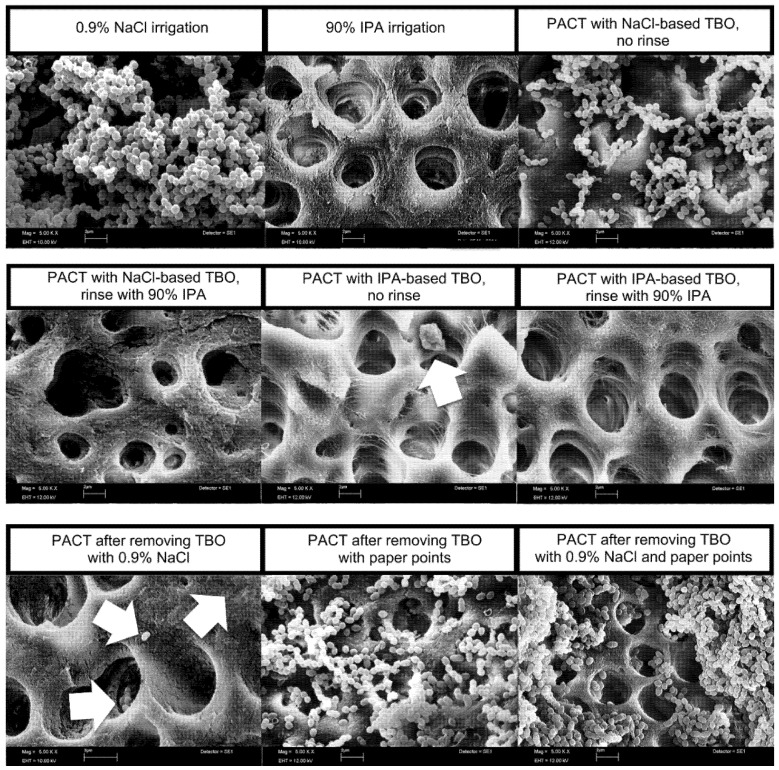
Scanning electron microscopic (SEM) images after different antimicrobial treatments (5000×). After irrigation using 0.9% NaCl, a biofilm-like structure of *E. faecalis* was found on the root canal surface and within dentin tubules. Arrows indicate bacterial cells and cell remnants on the root canal surface and within dentin tubuli.

Figure 3 shows representative SEM images of the root canal dentin for each treatment group. After rinsing root canals with 0.9% NaCl solution, a biofilm-like structure of *E. faecalis* was found. In the other treatment groups, a much lower amount of residue bacterial cells and debris was found on the root canal walls and within the dentinal tubules. Normal and abnormal shaped bacterial cells and cell remnants were found on the root canal surface and within dentinal tubules. Images of the root canal surface after antimicrobial treatments using PACT with NaCl-based photosensitizer and in groups, removing the photosensitizer with NaCl irrigation or paper points or both prior to light activation revealed some areas with large amounts of bacteria, as seen in the images, but the vast majority of the dentinal walls in these groups were free from microorganisms.

## 4. Discussion

Modifying the protocol of conventional PACT treatment in endodontics by removing excess photosensitizer before activating the photosensitizer with the light source significantly reduced *E. faecalis* counts in root canals artificially infected with *E. faecalis*. However, these modifications failed to significantly increase the antimicrobial effect of PACT against *E. faecalis* biofilm-like structures compared to conventional PACT. The idea behind this was that removing the excess photosensitizer in the root canals may increase the intracanal concentration of available oxygen and accordingly the concentration of cytotoxic oxygen derivatives necessary for antimicrobial effects of PACT. Furthermore, the dye (photosensitizing agents) is a dark blue solution. Light activation via the PACT system inside the root canals with the dark photosensitizer solution can limit the exposure of the emitted light into the biofilm-like structures. Another effect could have been that removing the excess photosensitizer can improve the spreading of the emitted light of the light source enhancing the effect of PACT [34]. However, removing the excess photosensitizer did not significantly increase the antimicrobial effect of PACT. The photosensitizing agent (TBO) may still not be able to penetrate in the deep layers of the bacterial biofilm-like structure sufficiently due to the presence of the biofilm matrix [27]. Additionally, the concentration of available oxygen in the bacterial biofilm in the root canals may still be too low for sufficient PACT effects. As a result, there may accordingly be a very low concentration of cytotoxic oxygen derivatives for PACT may accordingly be very low. Further, anatomic limitations, e.g., irregularities in the shape of the root canal systems, fins and the presence of dentinal tubules may be factors inhibiting the diffusion of the photosensitizer into the biofilm-like structure and within the dentin of the root canal walls. These conditions may either block or minimize the formation of cytotoxic oxygen derivatives and these areas may not be reached by the light during PACT [32].

Irrigants are used for chemomechanical root canal preparation and disinfection to reduce microorganisms to a minimum and dissolve and destroy the biofilm structure. Chelating agents, e.g., citric acid or ethylenediaminetetraacetic acid (EDTA) dissolve inorganic components of the biofilm [35]. In a previous study using photosensitizers based on citric acid and EDTA showed enhanced antimicrobial effects compared to conventional PACT [32]. Using a photosensitizer with 90% IPA as solvent significantly enhanced the antimicrobial effect of PACT against *E. faecalis* in the root canals in the present study. The antimicrobial effect of IPA has been investigated in detail in earlier studies [7,8,9,10]. Modifying the PACT protocol by combining the dye with 90% IPA could be favorable for the disintegration of the *E. faecalis* biofilm-like structure or rather destabilize superficial layers of bacteria. This could make PACT even more effective by enhancing the penetration of the photosensitizing agent [36]. Previous studies have shown that antimicrobial methods are more effective on planktonic microorganisms than on microorganisms organized in biofilms [37,38]. This emphasizes a possible role of IPA in enhancing the killing effects of the PACT using modified toluidine blue solution based on IPA.

In endodontic treatments, it is crucial to keep the elimination of microorganisms in the root canals to a minimum. Previous studies have shown that isolating culturable microorganisms after antimicrobial treatment indicate a poor prognosis for the tooth [39,40]. In the present study, dentin chips were collected after antimicrobial treatments of all groups to investigate the contamination of the dentinal tubules of the root canal walls. Conventional PACT, PACT followed by irrigation with IPA or irrigation with IPA alone significantly reduced *E. faecalis* viability in the root canals. However, these antimicrobial treatments failed to sufficiently penetrate into the biofilm-like structure or even into the dentin tubules, because no posttreatment sample in these groups was culture-negative. Only few culture negative root canals were found after PACT using IPA as solvent for toluidine blue and after conventional PACT when the photosensitizer was removed by paper points or by irrigation with NaCl solution and paper points. The highest portion of culture negative samples was found after antimicrobial treatment for PACT dissolving the photosensitizer followed by irrigation with 90% IPA (3 of 10 specimens). This group also had the highest reduction in *E. faecalis* counts after antimicrobial treatment. Using IPA as a solvent may enable the photosensitizer to penetrate into the innermost layers of the biofilm-like of *E. faecalis* leading to enhancement of the PACT effects. The additional isopropanol irrigation may further lower *E. faecalis* counts because PACT did already damage the biofilm-like structure and then the IPA can penetrate in deep layers of the biofilm to have additional antimicrobial effects.

The remaining contamination of the root canal dentin after antimicrobial treatment was visualized via SEM images as performed in a previous study [32]. SEM images depict the biofilm-like structures in the control rinsing root canals with 0.9% NaCl. After antimicrobial treatments with conventional PACT using NaCl-based photosensitizer and conventional PACT—when the photosensitizer was removed by NaCl irrigation and/or paper points—the root canals showed areas with numerous bacteria. In the other groups, bacterial cells and cell remnants were found on the root canal wall and in dentinal tubules. The remaining microorganisms inside the root canal system after chemomechanical preparation were associated with failure of the endodontic treatment [41,42].

*E. faecalis* was used for artificial root canal infections. It has been reported to be resistant against the bactericidal effects of intracanal dressings, like calcium hydroxide and antimicrobial agents, such as NaOCl, chlorhexidine and tetracycline [14,15,16].

Evaluating various disinfection protocols, *E. faecalis* has been used in several in vitro studies [28,32,43]. In the present study, *E. faecalis* formed a biofilm-like structure within 72 h of incubation as seen in previous studies [28,32]. The posttreatment SEM images of the root canal walls also show that *E. faecalis* is able to penetrate dentinal tubules, e.g., in the control (0.9% NaCl irrigation). In the present study, a monospecies biofilm using *E. faecalis* was used. *E. faecalis* was found as monospecies infection in root canals in previous in vivo reports [4,5]. Root canal infections are, however, often associated with multiple species [4,5,44]. Using a multiple species biofilm the content of different microorganisms may differ from specimen to specimen that may impair the effect of disinfection [3]. In the present study, sampling of the root canals was performed using sterile paper points as described in previous studies [27,30,32]. The paper points used for sampling have the appropriate size (ProTaper F2). The last endodontic file used for mechanical preparation of the root canal has the same size compared to the size of the paper points used for sampling. Thus, the paper points perfectly fit into the root canals for sampling. Nevertheless, the paper points are only able to take up a portion of bacteria from the root canal wall of the specimen. Additionally, sampling of dentin chips from the root canal walls was performed using a sterile ProTaper F3 hand file to examine the remaining contamination of the root canal dentinal surface. In this way, remaining bacteria on the dentinal wall and in dentinal tubules can be collected. Combining the two sampling methods the residue contamination posttreatment could be determined precisely. It also limits the underestimation of the bacterial contamination by sampling using paper points alone. Molecular techniques, like PCR techniques, are often used to detect microorganisms in root canals [4,5]. In the present study, the culture method was preferred, because in evaluating antimicrobial methods it was important to discriminate viable and nonviable bacterial cells. Bacterial contamination of the root canals after antimicrobial treatment would have been overestimated using molecular techniques [32]. Residual dead cells and cell remnants remaining in the root canals may contain DNA may lead to false positive results of the contamination, especially in treatment groups with PACT without irrigation. Irrigation techniques using antimicrobial agents with a certain flow rate will rinse off bacterial cells and cell remnants [32,45].

In summary, all treatments using PACT and/or IPA either as solvent for the photosensitizer or as irrigant after PACT significantly reduced *E. faecalis* viability in artificially infected root canals. Modifying the conventional protocol by removing excess photosensitizer before applying the PACT light source significantly reduced *E. faecalis* contamination in root canals but failed to significantly increase the antimicrobial effect of PACT against *E. faecalis* biofilm-like structures compared to conventional PACT using an NaCl-based photosensitizer. Dissolving the photosensitizer in 90% IPA significantly enhanced the antimicrobial effect of PACT against *E. faecalis* in the root canals. In addition, the final rinse with 90% IPA after PACT significantly increased the antimicrobial effect against *E. faecalis* biofilms compared to PACT treatment alone. However, no treatment has been able to completely eradicate *E. faecalis* in infected root canals. Even though the conditions in this in vitro study does not completely reflect the clinical situation, the effect of the different treatments on *E. faecalis* biofilm-like structure could be investigated in root canals. In this study, only teeth with one root canal were used. In teeth with more than one root canal and a more complicated root canal system anatomy results may be different. The dye and oxygen for PACT treatment and irrigants may not completely reach areas of the root canal system that are difficult to access, like fins, isthmi and lateral canals. However, using PACT with IPA-modified photosensitizer may be a promising alternative in cases when excessive irrigation may cause problems, e.g., wide open apices or perforations. The goal of root canal preparation and disinfection is a root canal system free from microorganisms. Future research on improving the efficiency of PACT may focus on the combination of PACT with root canal irrigants, e.g., NaOCl, chelating agents or alcohol in addition to dissolving toluidine blue in IPA.

## Supplementary Materials

The following are available online at https://www.mdpi.com/2079-6382/9/9/543/s1, Figure S1: Boxplots of CFU counts, demonstrating the antimicrobial effect of PACT on *E. faecalis* after 72 hour-incubation in root canals of extracted teeth (n = 270). Samples taken prior to treatment served as baseline, while sampling of the root canals using sterile paper points and collecting dentin chips after antimicrobial treatment were performed to determine the residual contamination of the root canals. A 16 µg/ml toluidine blue solution (TBO) served as the photosensitizer, activated by a 100 mW LED light source. The following treatment groups were tested: irrigation using 0.9% NaCl (negative control) or 90% isopropanol (IPA; positive control), conventional PACT with NaCl-based TBO and no final rinse (standard control), PACT with NaCl-based TBO and final rinse with 90% IPA, PACT with IPA-based TBO and no final rinse, PACT with IPA-based TBO and final rinse with 90% IPA, PACT with removal of access photosensitizer using either 0.9% NaCl solution or sterile paper points or both. Box plots represent the CFUs determined by selective agar plating, while horizontal lines indicate their median values. Undetectable values were ascribed the lowest detection limit value of the assay to allow for log transformation. Figure S2: Barplots (SD) of *E. faecalis* growth reduction (in %) following 72 hour incubation and PACT in root canals of extracted teeth (n = 270). A 16 µg/ml toluidine blue solution (TBO) served as the photosensitizer, activated by a 100 mW LED light source. The following treatment groups were tested: irrigation using 0.9% NaCl (negative control) or 90% isopropanol (IPA; positive control), conventional PACT with NaCl-based TBO and no final rinse (standard control), PACT with NaCl-based TBO and final rinse with 90% IPA, PACT with IPA-based TBO and no final rinse, PACT with IPA-based TBO and final rinse with 90% IPA, PACT with removal of access photosensitizer using either 0.9% NaCl solution or sterile paper points or both.
