# Supplementary material for: Improving the Efficiency of Photodynamic Chemotherapy in Root Canals against Enterococcus faecalis In Vitro
Source: Antibiotics (Basel). 2020 Aug 26;9(9):543. doi: 10.3390/antibiotics9090543 (PMC7560177; doi:10.3390/antibiotics9090543)
Supplement: Supplementary file 1 [file antibiotics-09-00543-s001.pdf]

Supplementary Material

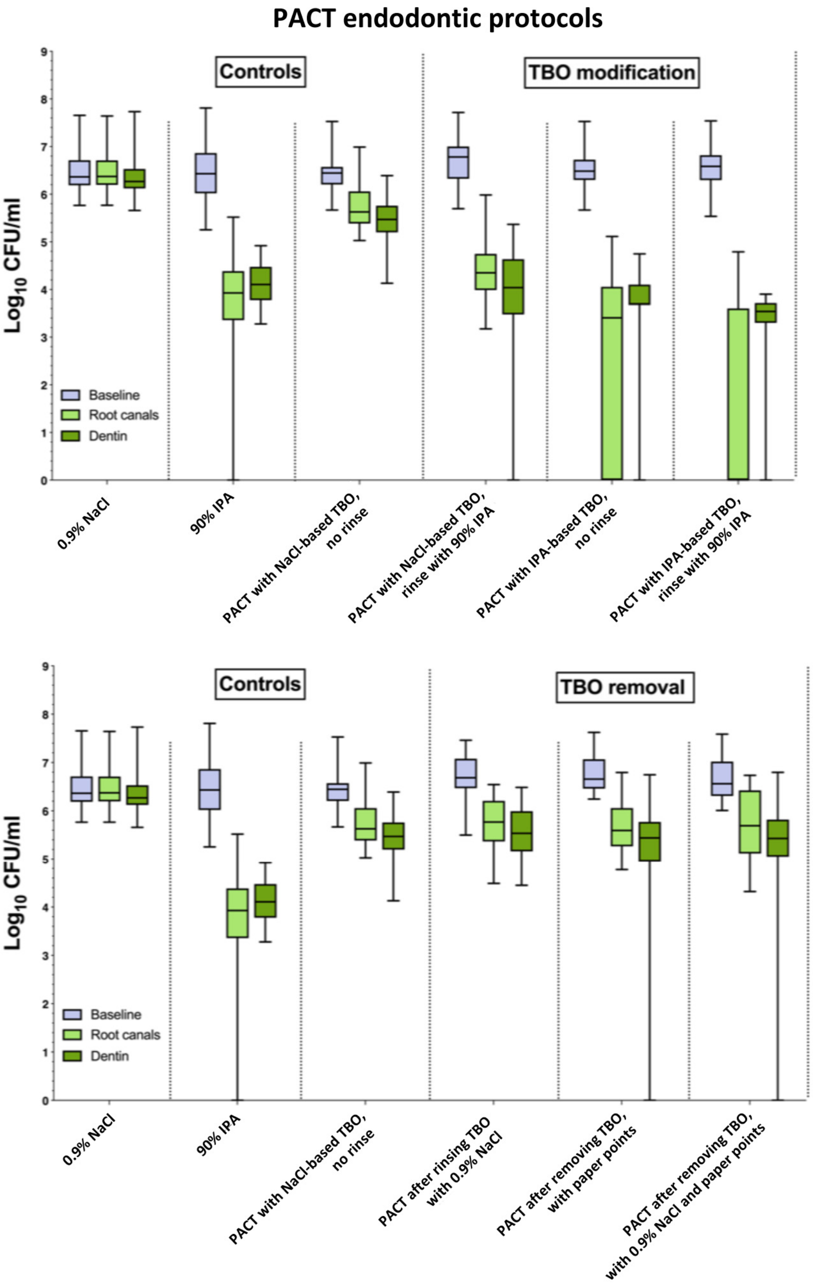

Figure 1. Boxplots of CFU counts, demonstrating the antimicrobial effect of PACT on *E. faecalis* after 72 hour-incubation in root canals of extracted teeth (n = 270). Samples taken prior to treatment served as baseline, while sampling of the root canals using sterile paper points and collecting dentin chips after antimicrobial treatment were performed to determine the residual contamination of the root canals. A 16 µg/ml toluidine blue solution (TBO) served as the photosensitizer, activated by a 100 mW LED light source. The following treatment groups were tested: irrigation using 0.9% NaCl (negative control) or 90% isopropanol (IPA; positive control), conventional PACT with NaCl-based TBO and no final rinse (standard control), PACT with NaCl-based TBO and final rinse with 90% IPA, PACT with IPA-based TBO and no final rinse, PACT with IPA-based TBO and final rinse with 90% IPA, PACT with removal of access photosensitizer using either 0.9% NaCl solution or sterile paper points or both. Box plots represent the CFUs determined by selective agar plating, while horizontal lines indicate their

median values. Undetectable values were ascribed the lowest detection limit value of the assay to allow for log transformation.

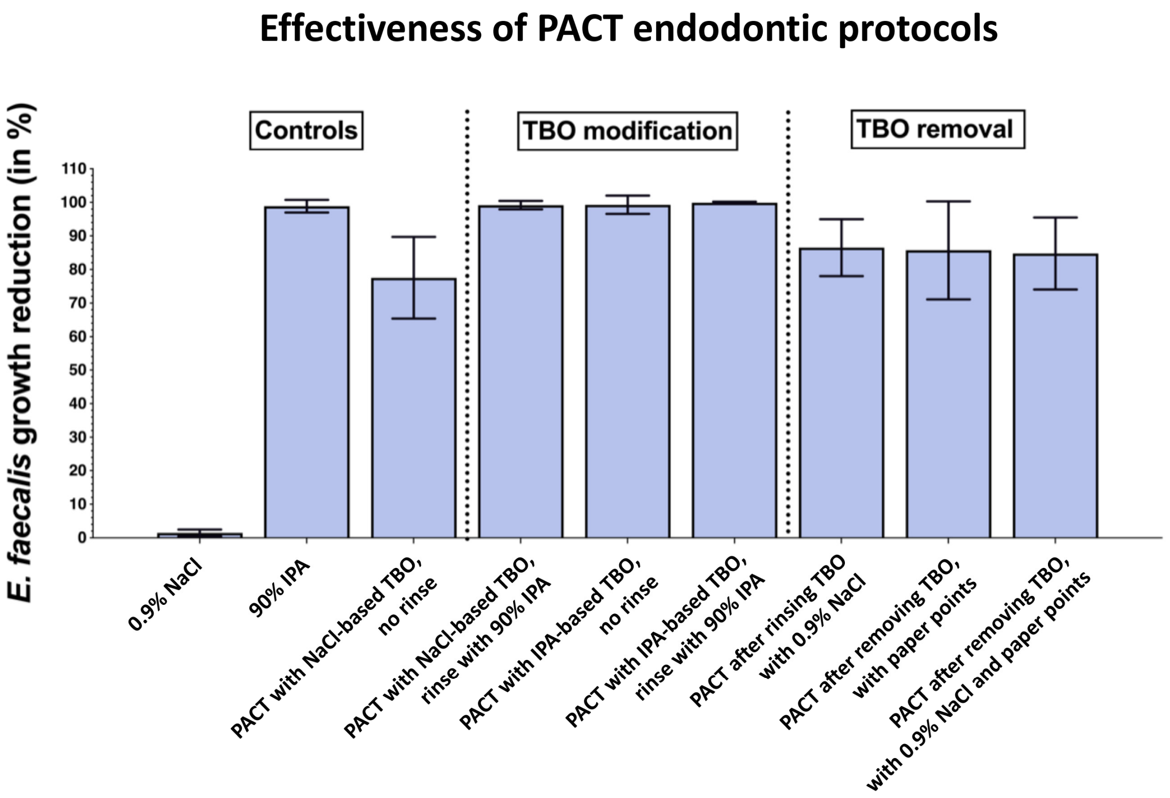

Figure 2. Barplots (SD) of *E. faecalis* growth reduction (in %) following 72 hour incubation and PACT in root canals of extracted teeth (n = 270). A 16 µg/ml toluidine blue solution (TBO) served as the photosensitizer, activated by a 100 mW LED light source. The following treatment groups were tested: irrigation using 0.9% NaCl (negative control) or 90% isopropanol (IPA; positive control), conventional PACT with NaCl-based TBO and no final rinse (standard control), PACT with NaCl-based TBO and final rinse with 90% IPA, PACT with IPA-based TBO and no final rinse, PACT with IPA-based TBO and final rinse with 90% IPA, PACT with removal of access photosensitizer using either 0.9% NaCl solution or sterile paper points or both.
